# Positive and negative psychosocial impacts on cancer survivors

**DOI:** 10.1038/s41598-023-41822-x

**Published:** 2023-09-07

**Authors:** Grace Yao, Jin-Shei Lai, Sofia F. Garcia, Susan Yount, David Cella

**Affiliations:** 1 Department of Psychology, College of Science, National Taiwan University, Taipei, 106319, Taiwan (R.O.C.); 2 Department of Medical Social Sciences, Department of Pediatrics, Robert H. Lurie Comprehensive Cancer Center, Feinberg School of Medicine, Northwestern University, 625 N Michigan Ave, 21St Floor, Chicago, IL 60611 USA; 3 Department of Medical Social Sciences, Department of Psychiatry and Behavioral Sciences, Center for Patient-Centered Outcomes, Robert H. Lurie Comprehensive Cancer Center, Feinberg School of Medicine, Northwestern University, Chicago, IL 60611 USA; 4 Department of Medical Social Sciences, Center for Patient-Centered Outcomes, Robert H. Lurie Comprehensive Cancer Center, Feinberg School of Medicine, Northwestern University, Chicago, 60611 USA

**Keywords:** Cancer, Psychology, Health care, Oncology

## Abstract

The purpose of this study is to understand psychosocial impacts on cancer survivors using the patient-reported outcomes measurement information system (PROMIS) Psychosocial Illness Impact banks. Cancer survivors (n = 509; age: 59.5 ± 1.4; 51.5% men) completed the PROMIS positive and negative illness impact items consisting of four sub-domains: self-concept (SC), social impact (SI), stress response (SR), and spirituality (Sp). Illness impact was defined as changed scores from items measuring “current” experiences to recalled experiences prior to cancer diagnosis. Descriptive statistics, effect sizes (ES), and coefficient of variation (CV) were calculated at item and sub-domain levels. Analysis of variance was used to identify potentially influential factors on the impacts. Our study found survivors reported stronger positive than negative impacts (overall ES mean: 0.30 vs. 0.23) in general; and more moderate (ES ≧ 0.30) positive than negative impacts at the item level, 54.3% (25 of 46) and 40% (16 of 40) for positive and negative items, respectively. Participants reported more positive impacts on SI and Sp but more negative impacts on SR. The CV results showed more individual differences appeared on positive SC items. Younger survivors reported stronger positive and negative impacts. Women reported higher positive impacts. Survivors with higher education levels tended to have higher positive SI impacts, while those with a lower family income reported higher negative SI and negative SR impacts. We conclude positive and negative psychosocial impacts coexisted—the strength of impacts varied across sub-domains. Age, gender, education, and family income influenced the psychosocial impacts reported by survivors. These findings provide a foundation to develop interventions to strengthen positive and minimize negative impacts and improve cancer survivors’ overall well-being.

## Introduction

With advances in cancer treatment, many cancers can now be controlled or managed for long periods^1^. Given the growing number of cancer survivors, understanding the psychosocial impacts of cancer and cancer treatment becomes critical to promoting survivors’ health-related quality of life. Historically, psychosocial research has focused on cancer’s negative consequences, such as mood disturbances, anxiety, cognitive problems, coping challenges, and interference with social relationships^2–10^. However, research also found that cancer experiences may increase personal resilience, which minimizes adverse impacts on illness^11, 12^. Survivors reported having greater life appreciation, changed priorities, closer relationships with families and friends, and enhanced spirituality after cancer diagnosis and treatment^13–22^. Thus, it is crucial to consider both the positive and negative impacts of the cancer experience^23^ in psychosocial interventions for cancer survivors by minimizing negative impacts yet enhancing positive impacts.

In 2004, the National Institutes of Health initiated a multicenter cooperative group called the Patient-Reported Outcomes Measurement Information System (PROMIS)^24^. PROMIS investigators developed reliable and valid measures of person-reported physical, mental, and social health, including psychosocial illness impact item banks^25^. The psychosocial illness impact item banks were developed using patient-centered approaches and items were generated based on interviews with cancer survivors. The initial psychometric evaluation results suggested that positive and negative illness impacts, although coexisting, were two independent constructs from a measurement perspective^26^. Subsequently, two different measures were recommended. Additional interviews with cancer survivors were conducted to ensure comprehensive content coverage, and the PROMIS Illness Impact Working Group wrote new items according to interview results. Items were classified into four sub-domains: self-concept (SC), social-impact (SI), stress-response (SR), and spirituality (Sp). To better capture the “impacts” of cancer diagnosis and/or treatment, patients provide two responses to each question: one is to consider the time before cancer diagnosis and/or treatment, and the other reflects the present. Field tests on patients with cancer showed that this measure has good reliability and validity^26–28^. The PROMIS psychosocial illness impact item banks are reported using an Item Response Theory (IRT) based T-score scoring matrix. The IRT-based T-scores consider varying degrees of discrimination and difficulty levels of each item on the measurement continuum, enabling a brief yet precise estimation of the construct of interest. Despite the well-known advantages mentioned above, our study showed individual items within the item bank might be more sensitive to individual attributes^29^. Patients with different types of cancer may have illness impacts in different aspects. For example, breast cancer survivors with a mastectomy and colorectal cancer survivors with an ostomy may have worse body image^30, 31^. These physical changes can affect cancer survivors not only in appearance but also in intimate relationships. Cancer survivors also face psychological and emotional issues, such as depression, grief, fear of recurrence, survivor guilt, etc^32, 33^. Understanding psychosocial impacts upon cancer diagnosis and treatment at the item levels and factors associated with those impacts can guide the development of personalized short forms by selecting items sensitive to change based on individual attributes.

This study explored the extent of psychosocial impacts since cancer diagnosis at the item and the sub-domain levels across the disease continuum. We sought to identify items exhibiting the most impacts, differences between negative and positive impacts, and factors associated with the impacts. The results of this study can help pave the way for the development of individualized interventions that strengthen the positive effects of cancer diagnosis and treatment and minimize the negative effects.

## Methods

We presented our study design and results following the reporting guideline from the EQUATOR Network^34–36^.

### Participants

Participants included 509 cancer survivors recruited from the Duke Cancer Care Research Program in Durham, NC (n = 72), the Duke Tumor Registry (n = 283), and NexCura, a nationwide online registry of more than 500,000 cancer survivors (n = 154). Survivors were eligible if they were 18 years or older, had a cancer diagnosis, and were fluent in English. This study was approved by the Institutional Review Board of Northwestern University, and all participants provided informed consent. All methods performed in this study follow the relevant guidelines and regulations.

### Measures

**Figure 1:**
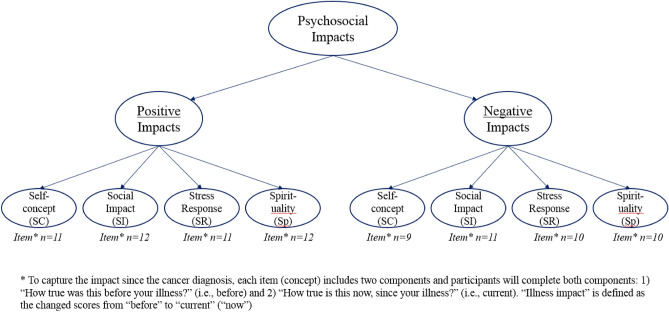
The structure of the psychosocial illness impact item banks.

This study was conducted as a cross-sectional study, and participants completed the following measures only once. The PROMIS Psychosocial Illness Impact item banks^27^ consist of four conceptual sub-domains (see Fig. 1 for the structure of the item banks): Self-Concept (SC), Social-Impact (SI), Stress-Response (SR), and Spirituality (Sp). Across these four sub-domains are 46 items measuring positive psychosocial illness impacts and 40 items measuring negative psychosocial illness impacts, including 11 positive and 9 negative SC items; 12 positive and 11 negative SI items; 11 positive and 10 negative SR items; and 12 positive and 10 negative Sp items (see Tables 1, 2 for item contents). Participants describe the extent to which the concept, as reflected in each item, affected their lives before their cancer diagnosis and/or treatment (How true was this *before* your illness?) and currently (How true is this *now*, since your illness?). In the following analyses, “before” referred to “How true was this *before* your illness?” and “current” referred to responses to “How true is this *now*, since your illness?” A 5-point rating scale is used: 0 = not at all, 1 = a little bit, 2 = somewhat, 3 = quite a bit, and 4 = very much. “Illness impact” was defined as the changed scores from “before” to “current” (“now”). Larger changed scores on positive and negative items indicated positive and negative impacts, respectively.

### Data analysis

Descriptive statistics were conducted at both item- and sub-domain levels on “illness impact” (“current”—“before”) (range: -4 to 4); that is, response to “How true is this *now*, since your illness?”—response to “How true was this *before* your illness?” Effect size (ES; mean of impact divided by standard deviation) was used to estimate the averaged magnitudes and direction of impacts. An ES is considered trivial when the absolute value < 0.1, small when ES is between 0.1 and 0.3, moderate when ES is between 0.3 and 0.5, and large when ES ≧ 0.5^37^. The coefficient of variation (CV; standard deviation divided by the impact mean) was used to estimate the variability of responses on individuals. An item with high variability implies its potential to capture a wide range of differences in individual responses to this item that are often overlooked during the averaging process^38^. Items with absolute CVs > 10 are considered to have significant variability^39^.

Analyses of variance (ANOVA) were used to investigate the potential factors associated with impacts, including age (< 50, 50–65, 65+), gender (male vs. female), ethnicity (White vs. non-White), relationship status (having vs. not, significant other including married, living with a partner), education (≦ high school vs. ≧ some college), and family income (< 20 K, 20–50 K, 50–100 K, > 100 K). Post-hoc (Tukey’s pairwise) comparisons were followed if overall a demographic variable was significant (*p* < 0.05).

### Consent to participate

Informed consent was obtained from all individual participants included in the study.

## Results

### Participants

Cancer survivors were primarily White (86.2%), married or living with a partner (75.4%), had at least some college education (84.2%), and had a household income higher than $50,000 (66.0%; 10.3% < 20 K, 23.7% 20–50 K, 37.6% 50–100 K, 28.4% > 100 K). They had an average age of 60.4 ± 11.4 years (20.4% < 50, 44% 50–65, 35.6% 65 +), 51.6% were male, 30.3% were full-time employed, and 36.2% were retired. Most survivors (87.5%) affiliated with a religion and expressed that religious affiliation was important to them (77.6%). Survivors had a wide range of cancer diagnoses, including breast (24.4%), colorectal (17.1%), prostate (15.7%), and lung (10.2%). The average time since cancer diagnosis was 4.7 ± 5.1 years (16.3% < 1 year, 32.3% > 5 years); 21.0% had experienced a cancer recurrence. Regarding treatment, 58.9% had received no treatment within the past month, 21.6% received chemotherapy, and 7.1% received radiation therapy. Most survivors reported normal daily activity with either no symptoms (54.0%) or some that did not require bed rest during the waking day (34.6%).

### Descriptive statistics

Descriptive statistics and CV and ES of impact scores are shown in Tables 1 and 2 for positive and negative measures, respectively. At the item level, means of impact scores were 0.26 (range − 0.10 to 0.66) and 0.23 (range − 0.54 to 0.77) for positive and negative items, respectively. For items capturing positive aspects, patients reported the largest change since cancer diagnosis on “*I see what is really important in my life*” (Sp12; impact score = 0.66), followed by ”*I am aware of the love and support available from other people*” (SI05; impact score = 0.62), and “*I am comfortable receiving help from others*” (SI07; impact score = 0.59). For those capturing negative aspects, patients reported the largest change since cancer diagnosis on “*I fear what will happen in the future*” (SR06; impact score = 0.77), followed by “*I worry about the future*” (SR01; impact score = 0.73) and “*Worry about my health interferes with my life*” (SR05; impact score = 0.73). At the sub-domain level, survivors reported almost no impact on positive SC (mean = 0.06) and negative Sp (mean = 0.07), yet large impacts on positive SI (mean = 0.39) and negative SR (means = 0.33). These results corresponded to the results at the item level. It was noted that patients reported minimal impacts on positive SC and negative Sp, where 10 (of 11) and 8 (of 10) items had an absolute impact mean≦ 0.2 for positive SC and negative Sp items, respectively.

**Table 1:** Descriptive statistics for impact scores on positive sub-domains (n = 509).

Item #	Item	Impact scores**		
		Mean(SD)	CV	ES
Overall mean(SD)		0.26(0.21)	0.38(15.9)	0.30(0.23)
*Self-concept*		0.06(0.59)^†^	− 8.34^‡^	0.07^‡^
SC01	I know I can handle difficult times	0.07(1.07)	15.29	0.07
SC02	I am comfortable with who I am	0.03(0.95)	31.67	0.03
SC03	I believe I can handle problems	-0.09(0.87)	− 9.67	− 0.10
SC04	I believe I am a confident person	-0.07(0.89)	− 12.71	− 0.08
SC05	I believe I am a good person	0.08(0.48)	6.00	0.17
SC06	I appreciate the health of my body	0.51(1.13)	2.22	0.45
SC07	I am an optimistic person	-0.02(0.85)	− 42.50	− 0.02
SC08	I can keep going when problems arise	-0.01(0.82)	-82.00	− 0.02
SC09	I can handle most anything	-0.09(0.92)	− 10.22	− 0.10
SC10	I believe I am a patient person	0.20(0.94)	4.70	0.21
SC11	I believe I am an honest person	0.07(0.38)	5.43	0.19
*Social impact*		0.39(0.57)^†^	2.41^‡^	0.47^‡^
SI01	I know who I can count on in times of trouble	0.33(0.90)	2.73	0.37
SI02	I have compassion for others	0.34(0.65)	1.91	0.52
SI03	I am comfortable asking others for help	0.53(0.99)	1.87	0.53
SI04	My relationships are meaningful	0.22(0.65)	2.95	0.34
SI05	I am aware of the love and support available from other people	0.62(0.97)	1.56	0.64
SI06	I realize who my real friends are	0.54(0.92)	1.70	0.59
SI07	I am comfortable receiving help from others	0.59(0.91)	1.54	0.64
SI08	I can appreciate people in my life	0.49(0.74)	1.51	0.66
SI09	I am willing to help others	0.20(0.69)	3.45	0.29
SI10	I make time for family and friends	0.36(0.86)	2.39	0.42
SI11	I feel connected to people in my community	0.20(0.94)	4.70	0.21
SI12	I feel close to people I care about	0.25(0.65)	2.60	0.39
*Stress response*		0.24(0.69)^†^	3.10^‡^	0.27^‡^
SR01	I am willing to express my emotions	0.42(0.83)	1.98	0.50
SR02	I am able to accept the way things work out	0.31(0.86)	2.77	0.36
SR03	I can deal with uncertainty	0.23(1.00)	4.35	0.23
SR04	I can adjust to things I cannot change	0.37(0.96)	2.59	0.39
SR05	I am able to take things as they come	0.31(0.91)	2.94	0.34
SR06	I am able to deal with stress and problems	0.06(1.03)	17.17	0.06
SR07	I tend to be accepting of things	0.33(0.91)	2.76	0.36
SR08	I take good care of myself	0.56(0.93)	1.66	0.60
SR09	I look at things in a positive way	0.16(0.89)	5.56	0.18
SR10	I am able to feel joy	0.00(0.94)	NA	0.00
SR11	I am able to enjoy life	-0.10(1.08)	− 10.80	− 0.09
*Spirituality*		0.32(0.66)^†^	4.08^‡^	0.37^‡^
Sp01	I can appreciate each day fully	0.43(0.98)	2.28	0.44
Sp02	My life is meaningful	0.12(0.91)	7.58	0.13
Sp03	I appreciate life	0.43(0.86)	2.00	0.50
Sp04	I have a strong faith	0.30(0.72)	2.40	0.42
Sp05	I have a sense of purpose in life	0.07(0.93)	13.29	0.08
Sp06	I feel peaceful	0.17(1.12)	6.59	0.15
Sp07	I find comfort in my faith or spiritual beliefs	0.32(0.77)	2.41	0.42
Sp08	I find strength in my faith or spiritual beliefs	0.32(0.76)	2.38	0.43
Sp09	I have a sense of peace	0.24(0.99)	4.13	0.25
Sp10	I feel close to God	0.35(0.79)	2.26	0.45
Sp11	I find strength in prayer	0.37(0.80)	2.16	0.46
Sp12	I see what is really important in my life	0.66(0.94)	1.42	0.70

SC, self-concept; SI, social impact; SR, stress response; Sp, spirituality; NA, not available.

^†^The bold value of Mean and SD for each sub-domain was calculated from survivors’ scores on the items within the sub-domain.

^‡^The bold values of coefficient of variation (CV) and effect size (ES) for each sub-domain were calculated from the mean of item scores within the sub-domain.

**Impact Scores: changed scores from before cancer to current.

**Table 2:** Descriptive statistics for impact scores on negative sub-domains (n = 509).

Item #	Item	Impact Scores**		
		Mean(SD)	CV	ES
Overall mean(SD)		0.23(0.27)	1.81(9.55)	0.23(0.25)
*Self concept*		0.24(0.67)^†^	4.57^‡^	0.27^‡^
SC01	I feel I am a failure	0.18(0.80)	4.44	0.23
SC02	I feel useless	0.35(0.94)	2.69	0.38
SC03	I feel that people do not respect me	0.06(0.69)	11.50	0.09
SC04	I feel worthless	0.23(0.83)	3.61	0.27
SC05	I feel inferior to others	0.13(0.74)	5.69	0.17
SC06	I am unhappy with my physical appearance	0.39(1.04)	2.67	0.38
SC07	I lack confidence	0.22(0.86)	3.91	0.25
SC08	I have a negative attitude toward myself	0.18(0.79)	4.39	0.23
SC09	I feel helpless	0.44(0.97)	2.20	0.45
*Social impact*		0.26(0.68)^†^	3.19^‡^	0.27^‡^
SI01	I feel like I am a burden to my family	0.56(1.09)	1.95	0.52
SI02	I have trouble asking others for help	− 0.21(1.00)	− 4.76	− 0.21
SI03	I feel isolated from others	0.31(0.99)	3.19	0.31
SI04	I feel disconnected from others	0.33(0.95)	2.88	0.35
SI05	I feel like a burden to others	0.48(1.02)	2.13	0.47
SI06	I have lost some close relationships	0.25(0.97)	3.88	0.26
SI07	I feel like people avoid me	0.25(0.75)	3.00	0.34
SI08	I feel guilty for being unavailable to family and friends	0.42(0.98)	2.33	0.43
SI09	It is hard for me to get close to people	0.16(0.84)	5.25	0.19
SI10	I have trouble relating to others	0.07(0.70)	10.00	0.10
SI11	I feel I need to hide how I really feel	0.19(1.00)	5.26	0.19
*Stress response*		0.33(0.66)^†^	0.21^‡^	0.29^‡^
SR01	I worry about the future	0.73(1.20)	1.64	0.61
SR02	I am bothered by little things	0.03(1.08)	36.00	0.02
SR03	I have difficulty accepting that things aren't always in my control	-0.03(1.01)	− 33.67	− 0.03
SR04	I get upset by small changes in my health	0.59(1.08)	1.83	0.55
SR05	Worry about my health interferes with my life	0.73(1.21)	1.66	0.60
SR06	I fear what will happen in the future	0.77(1.19)	1.55	0.64
SR07	I avoid thinking about my health	-0.14(1.21)	-8.64	− 0.11
SR08	I avoid going to the doctor	-0.54(1.13)	− 2.09	− 0.48
SR09	Worry about my health interferes with my sleep	0.65(1.12)	1.72	0.58
SR10	I get nervous before going to the doctor	0.57(1.17)	2.05	0.49
*Spirituality*		0.07(0.48)^†^	-0.87^‡^	0.11^‡^
Sp01	Difficult times weaken my faith	-0.04(0.58)	− 14.50	− 0.06
Sp02	I feel I have been given more than I can take	0.24(0.86)	3.58	0.28
Sp03	I am losing my faith	0.00(0.52)	NA	0.00
Sp04	I have trouble feeling peace of mind	0.24(0.93)	3.88	0.25
Sp05	My life lacks meaning	0.19(0.86)	4.53	0.22
Sp06	Difficult times weaken my spiritual beliefs	-0.04(0.51)	− 12.75	− 0.07
Sp07	My life lacks purpose	0.20(0.84)	4.20	0.23
Sp08	I question the purpose of my life	0.20(0.94)	4.70	0.21
Sp09	I feel punished my God	0.08(0.56)	7.00	0.15
Sp10	I find it hard to pray	− 0.07(0.59)	− 8.43	− 0.12

SC, self-concept; SI, social impact; SR, stress response; Sp, spirituality; NA = not available.

^†^The bold value of Mean and SD for each sub-domain was calculated from survivors’ scores on the items within the sub-domain.

^‡^The bold values of coefficient of variation (CV) and effect size (ES) for each sub-domain were calculated from the mean of item scores within the sub-domain.

**Impact scores: changed scores from before cancer to current.

### Standardized impacts—effect size

As shown in Tables 1 and 2, the overall ES means (SDs) were 0.30 (± 0.23) and 0.23 (± 0.25 for positive and negative impacts, respectively. Among all 86 positive and negative items, 26 (30.2%) items and 15 (17.4%) items had moderate or strong impacts (absolute ES value ≥ 0.3) towards a positive and negative direction, respectively. This suggests survivors generally reported more positive than negative impacts from their cancer experiences. At the sub-domain level, survivors reported small or moderate impacts towards a positive direction on positive SI (mean ES = 0.47), positive SR (ES = 0.27), and positive Sp (mean ES = 0.37). A negligible impact was reported on positive SC (mean ES = 0.07). Survivors reported small impacts towards a negative direction on all negative sub-domains: SC (mean ES = 0.27), SI (mean ES = 0.27), SR (mean ES = 0.29), and Sp (mean ES = 0.11). These results supported our previous findings that positive and negative impacts coexisted with different strengths across sub-domains^26^.

**Figure 2:**
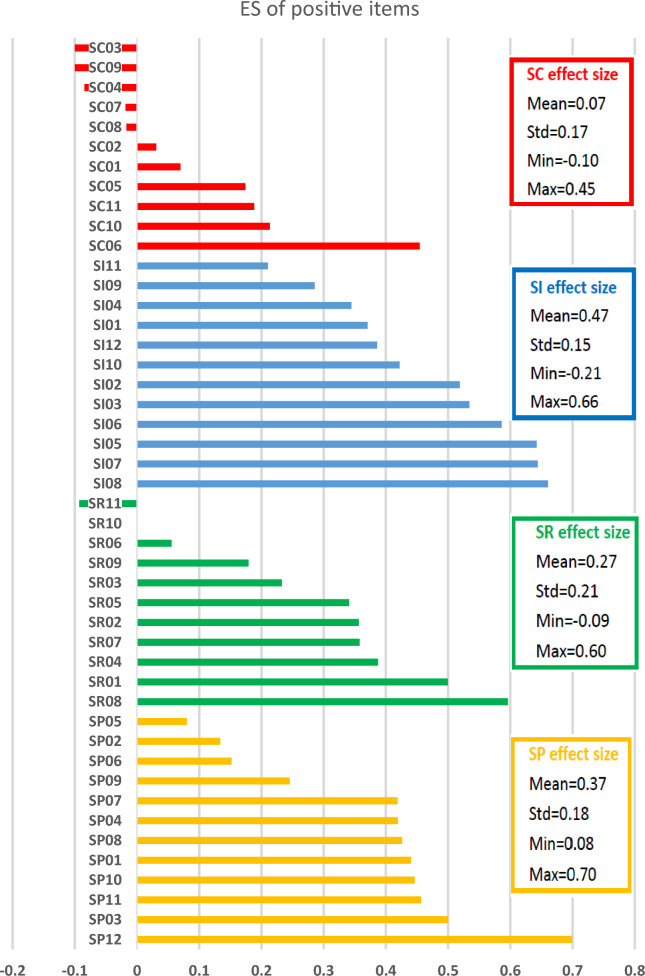
The effect size (ES) for positive items.

**Figure 3:**
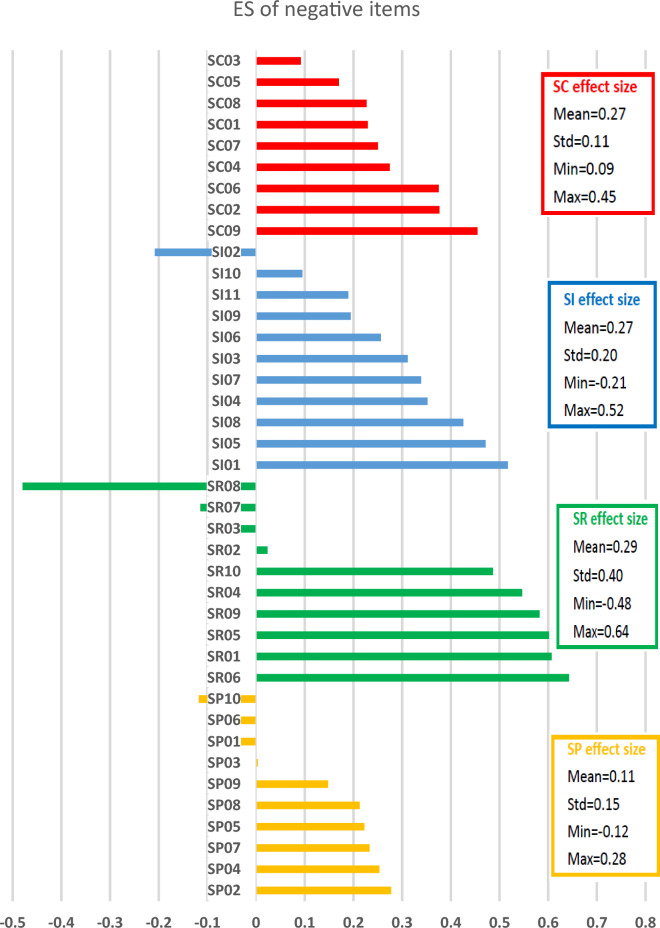
The effect size (ES) for negative items.

At the item level, for positive items, thirteen larger positive ES (see Fig. 2) suggested that survivors had more appreciation of their physical health (SC06, ES = 0.45), life (Sp03, ES = 0.50), important things in life (Sp12, ES = 0.70) and people in their life (SI08, ES = 0.66); found more love and support from others (SI05, ES = 0.64), more compassion (SI02, ES = 0.52), more strength in prayer (Sp11, ES = 0.46) and close to God (Sp10, ES = 0.45); realized who their friends are (SI06, ES = 0.59); they were more comfortable asking others for help (SI03, ES = 0.53), receiving help (SI07, ES = 0.64), and expressing emotion (SR01, ES = 0.50); and took better care of themselves (SR08, ES = 0.60) after the cancer diagnosis. For negative items, ten larger positive ES (see Fig. 3) indicated that survivors experienced greater worry (SR01, SR05, SR09, ES > 0.58), fear (SR06, ES = 0.64), helplessness (SC09, ES = 0.45), distress and nervousness (SR04, SR10, ES > 0.49), and feelings of guilt and being a burden (SI01, SI05, SI08, ES > 0.43) after their illness as compared to before.

### Variability of items

The variability of impact scores was small across all sub-domains with CVs < 10. It was noticed that 15 items (9 positive and 6 negative) had an absolute mean ≦ 0.1 but had an absolute CV greater than 10. Of these 9 positive items, 6 were positive Self-Concept items. These findings suggest significant individual variations on these items. However, changes in individual participants were inconsistent in direction resulting in the mean offsets impact changes in positive and negative directions.

### Influential factors of illness impact

Analyses of variance (ANOVA) showed significant results on age, gender, education, and income factors (*p* < 0.05). The following post-hoc comparison results are presented in Table 3. Age was a significant factor in 7 of 8 sub-domains across negative and positive impacts; younger survivors reported higher positive and negative impacts. Gender was a significant factor only in four positive subdomains but not in negative ones; women had higher positive impact scores than men. Survivors with higher education levels tended to have higher positive SI impact scores. Survivors with a lower family income had higher negative SI and negative SR impact scores. No significant effects were observed on ethnicity and relationship status.

**Table 3:** Post-hoc comparisons for analysis of variance on impact scores (n = 509).

	Positive sub-domains				Negative sub-domains			
	SC	SI	SR	Sp	SC	SI	SR	Sp
Age	1 > 3*	1 > 2 > 3	1,2 > 3	1,2 > 3	2 > 3	1 > 3	1 > 3	
Gender	2 > 1	2 > 1	2 > 1	2 > 1				
Ethnic								
Relation								
Education		2 > 1						
Income						1 > 4	1 > 2	

*1 > 3 represents category 1 had higher impacts than category 3.

All showed post-hoc comparisons are statistically significant with a p-value < 0.05.

Age: (1) < 50, (2) 50–65, (3) 65 +

Gender: (1) M, (2) F .

Ethnic group: (1) not white, (2) white .

Relationship: (1) with significant other, (2) no significant other .

Education: (1) ≦high school, (2) ≧some college.

Income: (1) < 20 K, (2) 20-50 K, (3) 50-100 K, (4) > 100 K.

SC, self-concept; SI, social impact; SR, stress response; Sp, spirituality.

## Discussion

The experience of cancer can be devastating but can also bring an opportunity for personal growth and new perspectives on life^40–44^. In this study, we examined the psychosocial impacts of cancer and its treatment using concepts raised by cancer survivors, which were then formatted to capture the psychosocial impacts due to cancer diagnosis in depth. Our results indicated positive and negative psychosocial impacts coexisted, coinciding with the literature^26, 40–45^. We suggest that interventions should consider both positive and negative impacts. Our findings can serve as a foundation to develop targeted, individualized interventions for whom increasing facilitators are needed by emphasizing positive impacts and minimizing barriers are needed by focusing on decreasing negative impacts.

Survivors reported different levels of impact upon contents addressed by individual items. A brief form that consists of items with large ES can be created for measuring survivor psychosocial impacts upon cancer if administering full-length PROMIS Psychosocial Illness Impact item banks or computerized adaptive tests is not feasible. Past research proposed three important facets of impact from severely stressful life events: self-perceptions, social relationships, and personal growth and life priorities^42–44, 46^. Tedeschi and Calhoun^47^ studied individual growth after encountering trauma such as cancer using Posttraumatic Growth Inventory. They found five factors: a greater appreciation of life, a changed sense of priorities, warmer and more intimate relationships, a greater sense of personal strength, and recognition of new possibilities or paths for one's life and spiritual development. Our results confirm these findings. Particularly, we found that survivors reported more social support (mean ES = 0.47 for positive items) and better spiritual well-being (mean ES = 0.37 for positive items) but had relatively more challenges with stress (mean ES = 0.29 for negative items) and worse self-concept (mean ES = 0.27 for negative items). These findings were also supported by Park and Blank’s study, in which cancer survivors reported larger positive impacts than negative ones^43^. However, their measure was not limited to psychosocial impact.

Individual differences were noted on 15 items with absolute CVs > 10, most related to positive SC. This result indicates that compared with other items, these SC items with larger CV reflected that the participants had more relative variation, either decrease or increase in their perception of "self" before/after the disease diagnosis. Yet the different directions of these item impact scores were canceled at the sub-domain level resulting in negligible impact scores. As items with larger CVs have the potential to discriminate against survivors with different levels of impact, these items could be considered candidates to detect individual differences over time. However, we should use CVs with caution. When the denominator (i.e., impact mean) is close to zero, the CV becomes very sensitive to small changes in the mean^48^. This condition occurs when participants’ impact scores show variation, but positive and negative scores offset the mean of impact scores.

Our results on age, gender, and income level are consistent with the literature^43, 45, 47, 49–55^. We found younger survivors reported greater life disruptions yet simultaneously reported a more positive attitude towards the disease. Female survivors reported a more positive attitude, but there is no gender difference in negative attitudes toward the disease. Survivors with lower income report more negative psychosocial impacts from cancer. However, the literature does not provide a consistent pattern about education, ethnicity, and relationship status in the psychosocial impacts of cancer^45, 50, 52, 54, 55^. Our study found survivors with higher education have a more positive attitude toward the disease. No significant differences were found in ethnicity and relationship status. Future studies on different sample groups should be conducted to evaluate the replicability of our findings.

This study had some limitations. Our sample was not nationally representative; there was an over-representation of well-educated and White survivors. Replication of our results with a more diverse set of survivors is needed. Another limitation is that we relied on survivors to recall their experiences before diagnosis, as it is not practical to conduct a prospective study, enrolling people before a cancer diagnosis. We attempted to minimize recall bias by implementing an appropriate question format by asking survivors to answer each item content with a before/after format; subsequently, the “before” question could be the reference for the “current” question. Our sample had an average time since cancer diagnosis of 4.7 years; thus, the recall accuracy was questionable. Future studies that evaluate psychosocial impacts at different stages of the disease continuum longitudinally, e.g., every year or every six months post-diagnosis, should be conducted to establish trajectory patterns of impact over time. In addition, we collected in this study from participants' self-reported perceived change, which might not be their veridical change. Boals and colleagues distinguished perceived and veridical stress-related growth and presented four possible constructs to shape perceived growth: (a) adherence to a cultural script, (b) reappraisal coping through secondary control or self-enhancement; (c) changes in narrative identity; and (d) violation of post-recovery expectations^56^. In other words, participants' perceived responses might be influenced by their cultures; they regulate their beliefs and reaction to fit the world's expectations by secondary control; they maintain personal continuity over time to face adverse experiences by autobiographical reasoning; and they attribute their experience by a convincing explanation. Perceived change may be a mix of these possible constructs. Future studies are needed to evaluate the replicability of our findings.

This study result may have implications for strategies in managing cancer survivors to promote their healthy adjustment to cancer throughout the disease continuum. The clinical focus of this research includes the impacts of cancer on self-concept, stress responses, social relationships, and spirituality; for example, to promote survivors’ quality of life after a cancer diagnosis, clinical practitioners can facilitate survivors’ positive consequences and alleviate negative ones.

In conclusion, this study expanded our prior work on the development of positive and negative psychosocial impact measures by requesting cancer survivors to report psychosocial impacts comparing before and after their cancer diagnosis^26^. Further, measures independently assessing the positive and negative psychosocial sequelae of illness allow for a more comprehensive measurement of how cancer affects individuals over time. Understanding these impacts sets the stage for developing interventions that can enhance the quality of life for survivors.

## Data Availability

The dataset can be accessed through the Healthmeasure Dataverse repository at https://dataverse.harvard.edu/dataverse/HealthMeasures.
